# Gli Proteins: Regulation in Development and Cancer

**DOI:** 10.3390/cells8020147

**Published:** 2019-02-11

**Authors:** Paweł Niewiadomski, Sylwia M. Niedziółka, Łukasz Markiewicz, Tomasz Uśpieński, Brygida Baran, Katarzyna Chojnowska

**Affiliations:** Centre of New Technologies, University of Warsaw, Banacha 2c, 02-097 Warsaw, Poland; s.niedziolka@cent.uw.edu.pl (S.M.N.); l.markiewicz@cent.uw.edu.pl (Ł.M.); t.uspienski@cent.uw.edu.pl (T.U.); b.baran@cent.uw.edu.pl (B.B.); k.chojnowska@cent.uw.edu.pl (K.C.)

**Keywords:** Hedgehog signaling, Gli proteins, posttranslational modifications, primary cilia, cancer, developmental signaling, morphogen signaling, nuclear import

## Abstract

Gli proteins are transcriptional effectors of the Hedgehog signaling pathway. They play key roles in the development of many organs and tissues, and are deregulated in birth defects and cancer. We review the molecular mechanisms of Gli protein regulation in mammals, with special emphasis on posttranslational modifications and intracellular transport. We also discuss how Gli proteins interact with co-activators and co-repressors to fine-tune the expression of Hedgehog target genes. Finally, we provide an overview of the regulation of developmental processes and tissue regeneration by Gli proteins and discuss how these proteins are involved in cancer progression, both through canonical regulation via the Hedgehog pathway and through cross-talk with other signaling pathways.

## 1. The Hedgehog Signaling Pathway

The hedgehog (Hh) pathway, discovered in Drosophila, is a highly evolutionarily conserved signaling cascade that orchestrates key steps of multiple aspects of development [1,2], including embryonic patterning, organ morphogenesis, and growth control by regulating cell proliferation, differentiation, and migration. The functions of the pathway depend on cell type, niche, and extracellular conditions [3,4].

Ligands from the Hh family are dually lipid-modified proteins with cholesterol and palmitate modification at their C and N termini [5], and can be locally secreted or diffuse over long distances [6,7,8]. Three ligands are produced in mammals: sonic hedgehog (Shh), Indian hedgehog (Ihh) and desert hedgehog (Dhh) [9]. Shh, is the most widely expressed isoform, and its loss is lethal for embryos [10,11,12]. Ihh regulates bone and cartilage development, whereas Dhh is essential for development in male gonads, peripheral nerves and the endothelium of large vessels [9].

Two transmembrane proteins are crucial for intracellular transmission of the Hh signal: the tumor suppressor membrane protein Patched (Ptch1) and the seven-transmembrane domain G-protein coupled receptor-like protein Smoothened (Smo) [13,14]. The activity of Smo affects the bifunctional glioma-associated oncogene transcription factors (Gli), which are transcriptional effectors of the Hh pathway. Gli proteins are members of the family of Kruppel-like factors with highly conserved Zn finger DNA-binding domains. In mammals, they are represented by three proteins: Gli1, Gli2 and Gli3. Gli1 acts principally as a transcriptional activator, whereas Gli2 and Gli3 display both activator and repressor functions [15,16,17]. 

**Figure 1:**
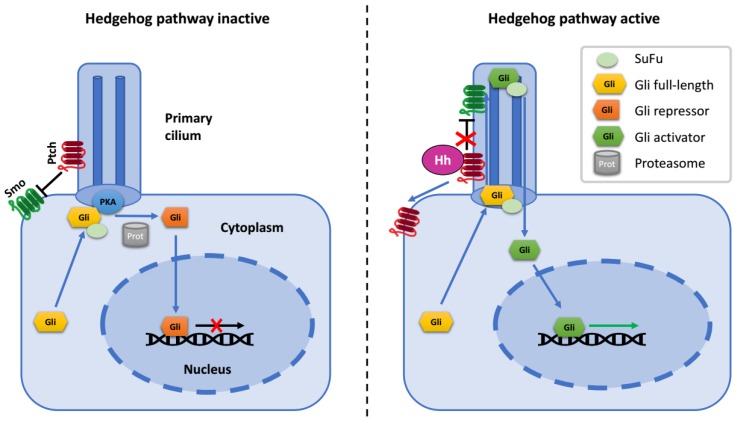
Schematic diagram of the hedgehog (Hh) pathway.

In the off-state of the Hh pathway, activity of Smo is inhibited by Ptch1. Under these conditions, Gli2 and Gli3 are partially truncated on the C terminus, which removes their transcriptional activation domain and confers on them the ability to function as repressors of Hh target genes. In addition to the creation of truncated Gli repressors, full-length Gli proteins are kept inactive through their interaction with the oncosuppressor SuFu (Suppressor of Fused), which restrains Gli in the cytoplasm and promotes its association with transcriptional corepressors [18,19] (Figure 1).

When Hh ligand binds to the Ptch receptor, it relieves the inhibition of Smo. Then, activated Smo blocks the proteolytic truncation of Gli proteins and promotes their dissociation from SuFu. Full-length Gli proteins undergo a series of posttranslational modifications and translocate into the nucleus, where they bind transcriptional co-activators and promote the expression of Hh target genes, including *Gli1* and *Ptch1* [4,14,18,19,20,21,22,23,24].

In mammalian cells, most components of the Hh pathway are situated at or in the proximity of the primary cilium—a tiny microtubule-based organelle which functions as a specific cellular antenna and is indispensable for Hh signaling [25,26]. Ptch and Smo are concentrated at primary cilium membrane: upon binding of the ligand, Ptch is excluded from the cilium, while Smo concentrates there. Smo accumulation at the ciliary membrane activates entry of SuFu and Gli proteins into the cilium [27]. Ciliary localization of Gli2 at the tip is required for its activation and its translocation to the nucleus as an activator of transcription [28]. What is more, the formation of the repressor forms of Gli also occurs in the vicinity of the primary cilium [29]. 

Anomalous activation or inhibition of Hh signaling is the leading cause of several diseases. Mutations in Hh pathway components, such as loss of function mutations of tumor suppressors Ptch and SuFu, or gain of function mutations of Smo or Gli proteins, have been discovered in many cancers [30,31,32,33,34,35,36]. In addition to cancer, aberrant Hh signaling in embryos results in developmental disorders such as holoprosencephaly, Greig syndrome, Ellis van Creveld syndrome, Pallister-Hall syndrome, peripheral neuropathy, osteoarthritis, cartilaginous neoplasia, and neurodegenerative diseases [37,38,39]. Aberrant activity of Hh signaling is also associated with many symptoms of so-called ciliopathies, that is, diseases caused by damaged or absent primary cilia.

Because the Hh pathway is involved in many disease states, being able to target it therapeutically is of high priority. The most common way of blocking Hh activity is by inhibiting Smo, and several Smo blockers are already approved for clinical use [40]. However, Smo inhibition can be overcome by developing resistance [41], and many cancers and developmental disorders are driven by Gli activation either downstream of Smo or through a cross-talk with other signaling pathways (see Section 8 and Table 2). Therefore, targeting Gli proteins directly would be the preferred therapeutic modality. Although several Gli inhibitors have been discovered [42], none are currently in clinical use. To design more effective inhibitors of Gli-dependent transcription, we must first understand the precise mechanisms of Gli regulation. In this review, we present the current state of knowledge about Gli protein function: their upstream regulators, posttranslational modifications, transport mechanisms, and ways in which they regulate target gene transcription. We also provide a survey of the involvement of different Gli proteins in development and disease in the hope of facilitating basic and translational research of these complex transcription factors. 

## 2. Gli Proteins

In Drosophila, Hh-regulated transcription is driven by a single transcription factor Cubitus interruptus (Ci), which acts both as an activator and repressor of transcription. In mammals, three homologs of Ci have been identified: Gli1, Gli2, and Gli3, each with specialized functions. 

**Figure 2:**
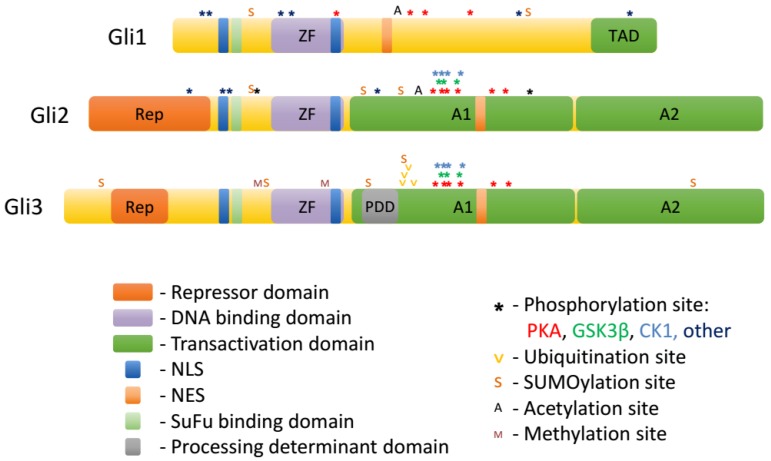
Domains and posttranslational modifications of Gli proteins.

Gli proteins belong to the GLI-Kruppel family of transcription factors. A hallmark of this group is the presence of C2H2-Kruppel-type zinc-finger (ZF) motifs in their DNA binding domains [16,17,43,44]. ZF domains of Gli1/2/3 bind the consensus sequence GACCACCCA [16]. The ZF domain of Gli proteins is localized centrally, with a shorter N-terminal domain upstream, and a longer C-terminal domain downstream. Whereas the sequence of the ZF domain is very highly conserved among the three Gli proteins, the different composition of N-terminal and C-terminal domains determine the specialized roles of each Gli protein (Figure 2.).

The N-terminal part of Gli2 and Gli3 harbors a repressor domain. Downstream of the repressor domain is a domain containing the proline-tyrosine (PY) nuclear localization sequence and the SuFu binding site (see Section 5) that are highly conserved across all three mammalian Gli proteins. This domain also contains putative activating phosphorylation sites in Gli proteins [23].

Downstream of the ZF domain resides the processing determinant domain [45]. The composition of this domain determines whether Gli proteins are proteolytically processed by the proteasome into truncated repressors (Gli3) or fully degraded (Gli1, Gli2). Further downstream is the phosphorylation cluster, which contains conserved PKA phosphorylation sites (P1-6 in Gli2 and Gli3, partially conserved in Gli1 [23,46]) as well as phosphorylation sites for GSK3β and CK1 (see Section 3). Phosphorylation of this cluster negatively regulates the ciliary and nuclear accumulation, as well as the transcriptional activity of Gli1/2/3 and triggers the formation of Gli3 repressor (Gli3R) through proteolytic processing. The area encompassing the processing determinant domain and the phosphorylation cluster was shown to possess transcriptional activation properties when fused to a heterologous DNA binding domain and was thus termed A1 [44]. However, when placed in the context of Gli2 N-terminus and ZF domain, A1 itself cannot mediate transcriptional activation. Another transcription activation domain termed A2 was also identified downstream of the phosphorylation cluster [44]. A1 and A2 domains are fairly well conserved between Gli2 and Gli3, but much less so in Gli1.

The domain composition of the three Gli proteins determines their function. Gli1 lacks the repressor domain and thus acts exclusively as a transcriptional activator. Gli3 contains an active processing determinant domain, and thus is efficiently processed into a C-terminally truncated Gli3R lacking the A1 and A2 domains. In most contexts, Gli3 is therefore primarily a transcriptional repressor, and its deletion results in most cases in gain-of-function phenotypes of Hh signaling. Gli2 has both a repressor domain and the two activator domains and could in principle function as both a repressor and an activator, but in practice mostly activates transcription of target genes, because processing of Gli2 into Gli2R is inefficient [45]. Importantly, Gli1 is typically not expressed in resting cells and is only produced upon stimulation of the Hh pathway via Gli2 and Gli3. Therefore, in the canonical Hh pathway, the level of transcription of Hh target genes is largely determined by the balance between Gli2 activator (Gli2A) and Gli3R. On the other hand, the expression of Gli1 can be stimulated non-canonically via cross-talk with other signaling pathways, which often happens in pathological states such as cancer.

## 3. Regulation of Gli Proteins by Posttranslational Modifications

Gli proteins are extensively modified posttranslationally, and these modifications largely determine their trafficking in the cell and ultimate transcriptional output of the Hh pathway. Among the posttranslational modifications of Gli1/2/3 are: phosphorylation, ubiquitination/proteasomal truncation, acetylation, sumoylation, methylation, and O-GlcNAcylation (Table 1 and Figure 2).

### 3.1. Phosphorylation

Phosphorylation is the best-studied modification of Gli proteins. Specifically, phosphorylation by protein kinase A (PKA) at sites P1-6 within the phosphorylation cluster is the key inhibitory signal regulating Gli proteins in the absence of the ligand [23]. P1-6 phosphorylation by PKA primes Gli2/3 for subsequent phosphorylation by casein kinase 1 (CK1) and Glycogen Synthase Kinase 3-β (GSK3-β) [46,54,55,73]. Phosphorylation of the first 4 of the P1-6 residues, and their cognate GSK3-β and CK1 sites, is sufficient to induce Cul1/β-TrCP-mediated ubiquitination and GliR formation (see Section 3.2). When the pathway is activated, dephosphorylation of P1-6 sites leads to the increase in Gli2A nuclear localization and transcriptional activity. For Gli1, sites that correspond to P1 and P2 (S544 and S560 in human Gli1) are also PKA targets and are required for restricting of Gli1 activity by PKA, even though Gli1 does not undergo PKA-dependent conversion into a repressor [48]. Thus, PKA is involved both in restricting full-length Gli1/2/3 activity and in priming Gli2/3 for ubiquitination and GliR formation [23,74]. 

Several kinases besides PKA have been shown to phosphorylate and regulate the activity of Gli transcription factors. Although CK1 plays a key role in GliR formation by phosphorylating PKA-primed sites within the P1-6 cluster, it is also involved in GliA formation and stabilization by protecting GliA from Cul3/Spop mediated proteasomal degradation [75]. 

Members of the dual specificity Yak-1 kinase family (DYRK) were shown to regulate Hh signaling at the level of Gli proteins. DYRK1a directly phosphorylates Gli1, which positively regulates Gli1 activity and increases its nuclear localization [56]. On the other hand, DYRK1b and DYRK2 exhibit negative regulatory function on Hh signaling [58,76]. While the precise mechanism by which DYRK1b regulates Hh signaling is not clear, it was shown that its expression correlates with downregulation of Hh target genes and transient Gli1 stabilization via the Akt pathway [57,76]. Finally, DYRK2 was shown to negatively regulate Hh signaling by direct phosphorylation of Gli2 at serine residues S385 and S1011, which targets Gli2 for proteasomal degradation [58]. 

ULK3 kinase directly phosphorylates all three Gli proteins, with Gli2 being the most phosphorylated [59]. Nuclear translocation of Gli1, but not Gli2 or Gli3, is increased by ULK3 phosphorylation. The association of ULK3 and Gli proteins is modulated by SuFu [77]. Interestingly, ULK3 is a close homolog of the Drosophila protein Fused, a positive regulator of Hh signaling in the fly.

Phosphorylation of Gli proteins is involved in the cross-talk between the mTOR pathway and the Hedgehog pathway. mTOR complex 1 (mTORC1) activation by TNFα results in Gli1 phosphorylation at serine residue S84 by S6K1 [60]. Phosphorylation of S84 prevents Gli1 interaction with SuFu allowing for Gli1 translocation to the nucleus. On the other hand, mTORC2 appears to be involved in Igf-mediated induction of Gli proteins in osteoblasts. Gli2 is phosphorylated on S230 by mTORC2-dependent kinase Akt, which stabilizes Gli2 and results in stronger induction of Hh target genes by a Smo agonist purmorphamine [62]. Akt-mediated phosphorylation of S230 is also involved in non-canonical Smo-independent induction of Gli2 in a mouse model of pancreatic cancer, which results in increased TGFα expression [61]. Interestingly, S230 in Gli2 corresponds to S84 in Gli1, the serine residue phosphorylated by S6K1 in TNFα-stimulated cells [60]. This serine residue is also localized within the Pc-g serine/threonine cluster identified as positively regulating Gli protein activity upon phosphorylation [23].

In basal cell carcinomas (BCC), atypical protein kinase C ι/λ (aPKC) positively regulates Gli1 by phosphorylating two serine/threonine residues S243 and T304, which strengthens GLI1 DNA binding. Pharmacological inhibition of aPKC suppresses the growth of SMO-inhibitor resistant tumors [49]. APKC-dependent phosphorylation acts primarily by targeting Gli1 for deacetylation by HDAC1 [50,78].

AMP-activated protein kinase (AMPK), which is a cellular metabolic sensor, phosphorylates GLI1 at residues S102, S408, and T1074 residues. This results in GLI1 destabilization and downregulation of Hh-dependent transcription [51].

MEKK1 kinase phosphorylates Gli1 at multiple residues in the C-terminal domain and blocks Gli1 transcriptional activity. Interestingly, two of the sites identified as potential MEKK1 targets overlap with targets for PKA, a known inhibitor of Gli proteins [52].

In breast cancer cells, the kinase CIT induces Gli2 phosphorylation at S149 and its nuclear translocation, with consequent upregulation of transcription of genes involved in cancer invasiveness [63].

Although most identified phosphorylation events on Gli proteins are mediated by serine/threonine-kinases, the tyrosine kinase Hck was recently shown to phosphorylate multiple tyrosines in human Gli1, including Y800 and increase the ability of Gli1 to induce target genes [53]. Hck also induces the dissociation of Gli1 and Gli2 from SuFu, but this latter effect appears independent of its kinase activity. Interestingly, Hck itself is a Gli1 target gene, contributing to a positive feedback loop between the two proteins.

In addition to phosphorylation by known kinases, there is evidence for Gli protein phosphorylation events where the kinase has not been identified. Upon pathway activation, Gli3 exhibits a unique phosphorylation pattern in the nucleus, which results in a shift in apparent molecular weight [19]. Activated Gli2 was also shown to be phosphorylated at S248 within a serine/threonine-rich cluster Pc-g, but the kinase responsible for this event remains to be discovered [23].

In contrast to the many protein kinases that have been identified as directly regulating Gli activity, few reports describe enzymatic dephosphorylation of Gli transcription factors by protein phosphatases. Gli3 is negatively regulated by protein phosphatase 2A (PP2A). Inhibition of PP2A increases nuclear translocation and transcriptional activity of Gli3 [79]. However, whether the effect of PP2A on Gli3 is direct and what phosphorylation sites on Gli3 are affected remain unknown. Conversely, the activity and nuclear accumulation of Gli1 is positively affected by the protein phosphatase WIP1, but whether the mechanism is through direct dephosphorylation of Gli1 is currently unclear [80].

### 3.2. Ubiquitination

Gli protein ubiquitination and proteasomal processing play a role both in GliR formation and GliA degradation, keeping the pathway in the off-state and providing a negative feedback loop in the on-state. Several E3 ligase complexes catalyze the addition of ubiquitin chains to lysines in Gli proteins. Two of these E3 complexes have been extensively characterized: Cul1/β-TrCP and Cul3/Spop.

Sequential PKA/GSK3β/CK1 phosphorylation allows for Gli2/3 interaction with the Cul1/β-TrCP complex. Interaction with Cul1/β-TrCP results in Gli3 ubiquitination and subsequent proteolytic processing leading to the formation of GliR [55]. The processing of Gli3 resembles partial proteasomal degradation of proteins such as NF-kB precursors p105 and p100, and the yeast proteins SPT23 and MGA2 [81]. Cul1/β-TrCP also ubiquitinates Gli1 and Gli2, but these two proteins are not typically processed, but rather completely degraded [64,82]. Whether a Gli protein is degraded or processed as a consequence of Cul1/β-TrCP-mediated ubiquitination is determined by the Processing Determinant Domain upstream of P1-6 phosphorylation sites [45]. 

The interaction between Gli1/2/3 and Cul1/β-TrCP is dependent on the presence of conserved DSG(X)2+nS degron motif. Also, Gli2 phosphorylation of residue S662 within that degron appears necessary for efficient interaction with β-TrCP [64]. Similarly, phosphorylation of Gli3 at serine residues S894 and S899 in a different DSG(X)2+nS β-TrCP degron was reported to be necessary for Gli3 processing to Gli3R [46]. Interestingly, when tested at endogenous expression level in NIH-3T3 Flp-In system, S894/S899 double mutation strongly reduced, but did not completely abolish Gli3R formation (P.N. data not shown). Another level of complexity was added when Gli3 was reported to possess multiple regions interacting with β-TrCP and multiple lysine clusters being ubiquitinated. As it turns out, only the mutation of 4 lysine residues K773, K779, K784 and K800 upstream to P1-6 PKA phosphorylation sites is sufficient to inhibit Gli3 processing to the repressor form [46].

As opposed to Cul1/β-TrCP, which ubiquitinates Gli proteins in their inactive state, another E3 complex Cul3/Spop is involved in the degradation of activated Gli proteins. It is unclear which Gli proteins are sensitive to Cul3/Spop-induced proteasomal degradation in vivo. Cul3/Spop interacts with Gli2 and Gli3 full-length proteins as well as Gli3R, and increased expression of Spop correlates with a decrease in Gli2/3 full-length but not Gli3R protein levels in C3H10T1/2 cells [65]. A similar observation was made in Drosophila S2 and gastric cancer cell lines [83,84]. However, another study reported that mouse Spop homozygous mutants show increased levels of Gli3 full-length and Gli3R but have normal levels of Gli2, suggesting that Gli2 degradation is not dependent on Cul3/Spop-mediated ubiquitination [85]. Spop mutants exhibit decreased Ptch1 mRNA level consistent with Hh pathway downregulation. Also, in the cerebellum, deletion of Spop increases Gli2 protein levels, but does not contribute to increased Gli2-mediated proliferation of granule cell progenitors or induce tumor formation, unless combined with SuFu deletion [86]. Additionally, contrary to the observation in C3H10T1/2 cells, increased Spop expression in HEK293T cells correlates with decreased level of Gli3 1-700 (mutant resembling Gli3 repressor) [65,85]. Therefore, more research is needed to unravel Cul3/Spop role in the regulation of full-length and repressor forms of Gli2/3 in vivo.

Unlike Gli2 and Gli3, Gli1 is not a target for Cul3/Spop. Its degradation is instead mediated by Numb, an adaptor protein which recruits it to E3 ligase Itch, leading to Gli1 degradation [84,87,88]. In addition to Itch, P300-associated factor PCAF was shown to regulate Gli1 expression and activity by its ubiquitination and subsequent proteasomal degradation in response to genotoxic stress [89]. PCAF is a somewhat peculiar player in Hh pathway regulation; its acetylation activity is needed for Hh target gene expression [90]. Indeed, PCAF interaction with Gli1 was positively correlated with histone 3 acetylation at Hh target gene promoters, which is a hallmark of actively transcribed genes. 

Proteasomal proteolysis of ubiquitinated Gli proteins can be inhibited by removal of the ubiquitin modification by enzymes known as deubiquitinases. The role of Gli protein deubiquitination has only recently come to light. The deubiquitinating enzyme HAUSP, a homolog of drosophila USP7, was shown to interact with all three Gli proteins [66]. HAUSP protects Gli2/3 and Gli1 from Cul3/Spop- and Itch-mediated proteasomal degradation, respectively. Gli1 ubiquitination was shown to be also regulated by USP48 [67]. USP48-mediated Gli1 deubiquitination stabilizes Gli1 protein and increases its activity. USP48 provides a positive feedback loop in Hh signaling as USP48 expression is induced by Hh activation. Another deubiquitinase USP21 was shown to negatively regulate Hh signaling [91]. Although USP21 stabilizes Gli1, it also recruits Gli1 to the centrosome where it promotes its phosphorylation by PKA, making it inactive. USP21 also interacts with Gli2, suggesting that it may have a similar effect on Gli2/3. Finally, the deubiquitinase OTUB2 was reported to interact with and stabilize Gli2 [92].

### 3.3. Sumoylation

Sumoylation is a modification that resembles ubiquitination and is catalyzed by enzymes that function similarly to ubiquitin ligases. In the off-state Gli2 undergoes sumoylation at lysine residues K630 and K716 [69]. Gli2 sumoylation is reduced upon Hh pathway stimulation or in the constitutively active Gli2 P1-6A mutant, suggesting that it negatively regulates Gli activity. Consistently, Gli2 harboring mutation in K630 and K716, which cannot be sumoylated, shows increased transcriptional activity and is unable to bind HDAC5, a negative regulator of gene expression.

In contrast, the Pias1 SUMO ligase appears to increase the transcriptional activity of Gli proteins. It directly sumoylates Gli1/2/3, which correlates with decrease in Gli protein ubiquitination, thus protecting them from proteasomal processing or degradation [68]. Pias1 overexpression increases Gli-dependent transcription and ectopic expression of Nkx2.2 in the chick neural tube, a marker of ectopic activation of Hh-dependent transcription.

Sentrin-specific peptidase (SENP1) was shown to specifically desumoylate Gli1 and negatively regulate Hh signaling. SENP1-mediated desumoylation promotes Gli1 ubiquitination and facilitates Gli1 nuclear export [70].

### 3.4. Acetylation

Histone acetyltransferases are important components of transcriptional activation complexes. In addition to acetylating histone tails, these enzymes have many other substrates. In particular, Gli1 and Gli2 are acetylated, and their acetylation/deacetylation constitutes an important functional switch. Gli1 and Gli2 are acetylated by the acetyltransferase p300/CBP at conserved lysine residues K518 and K757 respectively [24]. Acetylation at these residues reduces the ability of Gli proteins to induce target gene transcription by sequestering inactive Gli1 at the nuclear lamina and preventing both DNA binding and exportin 1-mediated nuclear export [50]. HDAC1-mediated Gli1 deacetylation in response to Shh and aPKC-dependent phosphorylation increases pathway activity. Conversely, Hh signaling is quenched when HDAC1 activity is blocked by siRNA, specific inhibitors, or induction of its proteasomal degradation [24,93]. REN/KCTD11 is an adaptor for Cul3 E3 ligase which recognizes HDAC1 and targets it for degradation [24]. Shh induced HDAC1 expression and Gli1 deacetylation provides a positive feedback loop whereas Cul3-REN/KCTD11 dependent HDAC1 ubiquitination and subsequent degradation provide negative pathway regulation [24].

### 3.5. O-GlcNAcylation

Gli1 and Gli2 may become modified by O-linked β-*N*-acetylglucosamine (O-GlcNAc). This modification enhances their nuclear accumulation and transcriptional activity and may be critical in mediating hyperglycemia-induced drug resistance in breast cancer [71].

### 3.6. Methylation

Gli3 was recently shown to be methylated at lysines K436 and K595 by the methyltransferase Set7, which positively regulates its stability and DNA binding ability [72]. In contrast, activities of Gli1 and Gli2 are not affected by Set7 even though K436 is conserved in Gli2 and K595 is conserved in all three Gli proteins.

## 4. Regulation of Gli Proteins at Primary Cilia

The primary cilium acts as an area for the integration of proteins that mediate processing and modifications of Gli transcription factors, including most canonical components of the Hh pathway. Gli proteins cannot function as activators or be processed into repressors in cells with defective intraflagellar transport (IFT) machinery, which is essential for the construction and proper functioning of the cilium. IFT mutant mice show developmental phenotypes characteristic of defective GliA and Gli3R [26,94,95]. Gli proteins themselves translocate into the primary cilium within a few minutes from Hh signal reception [22,96]. Several lines of evidence suggest that primary cilia are not only important for upstream Hh signaling via Ptch1 and Smo, but that they are directly involved in the conversion of Gli proteins into GliA and GliR downstream of Smo.

Conversion of Gli proteins into GliR, as well as inhibition of GliA formation, is associated with their interaction with two key negative regulators of the Hh pathway: SuFu, and PKA. SuFu sequesters Gli2/3 in the cytoplasm and blocks Gli protein transcriptional activity in the nucleus [19,97,98]. On the other hand, PKA phosphorylates Gli proteins, promoting the formation of GliR and limiting the transcriptional activity of full-length Gli proteins [23]. Interestingly, both SuFu and PKA are associated with the primary cilium in Hh-responsive cells. SuFu translocates into the cilium with Gli proteins [22] and the rapid dissociation of SuFu from Gli, which leads to the nuclear entry of GliA, appears to take place inside the cilium [19,20]. Consistent with the role of cilia in Gli-SuFu dissociation, when SuFu is lost, the transcription of Gli target genes is elevated independently of cilia [18,99].

Negative regulation of Gli proteins by PKA also occurs at the primary cilium. PKA subunits Cα, RIα and RIIβ localize to the ciliary compartment [100]. Inactive PKA accumulates at the base of the cilium upon pathway activation by Shh, while Shh withdrawal activates this periciliary PKA pool, but not total cellular PKA [74,101]. PKA is tethered to the periciliary compartment via the PKARIIβ subunit by the centrosomal protein Talpid3 and mutation of this protein results in reduced Gli2/3 phosphorylation and repressor formation [102]. Gpr161, a ciliary G-protein coupled receptor and negative regulator of Gli proteins, is essential for maintaining high ciliary PKA activity in the absence of Hh signal [103]. 

Epistasis experiments show that, unlike SuFu, PKA lies upstream of cilia in the Hh signaling cascade; cells that lack cilia can no longer induce Hh-dependent transcription by deactivation of PKA [29,104]. Finally, in addition to regulating Gli proteins directly, PKA may control the ciliary localization and function of the Gli/SuFu complex by phosphorylating SuFu [105]. 

### Gli Protein Transport to the Primary Cilium

**Figure 3:**
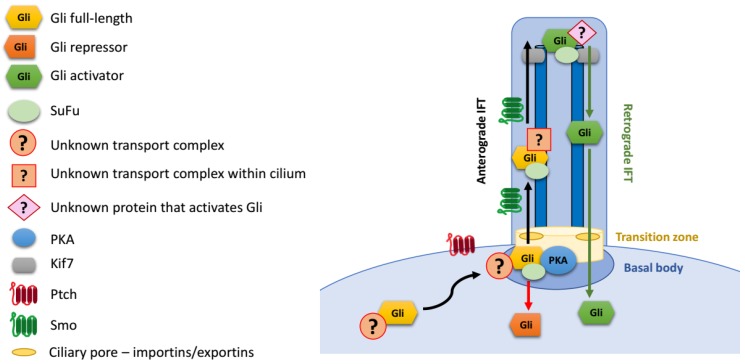
Gli protein transport to the primary cilium.

The mechanism of transport of Gli proteins into cilia remains incompletely understood (Figure 3). Gli1/2/3 are large proteins that diffuse slowly, and the entry of soluble proteins into the cilium is limited not only by the small surface area of the “ciliary pore”, but also by its properties as a diffusion barrier [106,107,108,109]. Moreover, the accumulation of Gli proteins at cilia tips, which occurs on the time scale of a few minutes, is further hindered by the crowded environment inside the cilium. Overall, the transport of Gli proteins to the cilium tip must consist of three steps: (1) targeted transport to the cilium base; (2) gated entry of proteins into cilia through a diffusion barrier; and (3) active trafficking within cilia towards the tip.

Little is known about how soluble Gli transcription factors are efficiently trafficked from the cytoplasm to the cilium base (step 1). For vesicle-embedded membrane proteins, the trafficking to the cilia from the Golgi on endosomal pathway has been well described [110,111]. However, Gli proteins are soluble rather than membrane-embedded, and the mechanism of their transport to the cilium base is likely to differ from that of transmembrane receptors, such as Ptch1 or Smo.

Translocation through the diffusion barrier between the basal body and the cilium (step 2) is another important step in Gli trafficking. The transition zone at the base of cilia forms a molecular sieve, which affects the entry of soluble proteins into cilium in a size-dependent manner [106,108,109]. The transport through this molecular sieve is thought to resemble the nuclear import/export of soluble proteins through the nuclear pore: smaller proteins can passively enter the cilium by diffusion, while larger proteins must be actively ferried through the diffusion barrier [106,108,111]. Importantly, the active transport through the ciliary pore involves some of the same proteins as those involved in nuclear import: Ran and importins/karyopherins. Specifically, karyopherin β2 (Kapβ2) regulates ciliary localization of Gli proteins and then their activation [112]. The interaction of Gli2 with Kapβ2 and its ciliary localization is dependent on the PY-NLS sequence that was initially identified as a nuclear localization sequence. Importantly, Gli3R formation is not affected in cells depleted of Kapβ2, suggesting that Gli3 processing may not require the passage through the ciliary diffusion barrier [112].

Upon entry into the cilium, Gli transcription factors strongly accumulate at its tip [22,113]. The canonical mode of transport from the base of the cilium to the tip involves the IFT machinery, driven by kinesin motors. The atypical kinesin Kif7 has been implicated in tip accumulation of Gli proteins. It interacts with Gli proteins [114] and, like them, localizes at the tip of the cilium [115]. Even though it is a molecular motor, Kif7 plays a key role in retaining Gli proteins in the tip compartment by organizing microtubules rather than actively transporting Gli1/2/3 to the tip [116,117]. 

Anterograde IFT components Ift172 and Ift88 are required for the activation of the targets of target genes by Gli transcription factors [95], but whether their role is direct or indirect remains unknown. Interestingly, depletion of Ift172 in the retina impaired ciliary localization of Gli1, but not Gli2, Gli3, or Smo [118]. Deficiency of another anterograde IFT component, Ift27, impairs Hh pathway signaling resulting in atypical ciliary trafficking of Smo and Gli2, and defective processing of Gli3 [119,120]. Gli2, SuFu and Kif7 enter the cilia in Ift27 mutant cells, but are no longer concentrated at cilia tips. This effect appears to be at least partially independent of Smo trafficking [119,121]. Importantly, unlike defects in Ift172 or Ift88, loss of Ift27 does not impair ciliogenesis. 

The IFT transport machinery travels alongside microtubules that build the cilium axoneme. Ciliary microtubules are highly glutamylated, but until recently the role of axonemal tubulin glutamylation was not clear. Interestingly, either defects of tubulin glutamylation or forced dynamic deglutamylation in the cilia impair the tip accumulation of Gli3 [122,123]. Whether the effect is due to slower anterograde IFT [123] or is IFT-independent [122] remains somewhat controversial.

The mechanisms of ciliary trafficking of Gli proteins rely on the interaction of components of different transport complexes with specific domains within Gli proteins themselves. The translocation of Gli proteins through the ciliary pore is mediated by the interaction of Kapβ2 with PY-NLS located near the SuFu-binding domain. However, PY-NLS is not sufficient to mediate the ciliary tip accumulation of the Drosophila Gli homologue Ci [112]. An additional domain necessary for the ciliary localization of Gli proteins has been located within the A1 activation domain [28,113]. It remains to be determined whether a combination of this domain with the PY-NLS is sufficient to localize proteins to the primary cilium tip, or whether additional protein sequences are necessary. Also unknown is the mechanism by which the identified A1 subdomain promotes Gli ciliary trafficking.

## 5. Nuclear Transport of Gli Proteins

Dynamic bidirectional transport between the cytoplasm and the nucleus is critical for the regulation of many transcription factors, whose levels inside the nucleus must be tightly controlled. Nucleocytoplasmic shuttling of proteins is mediated by members of the karyopherin family of proteins, such as importin α, importin β1, importin β2, and chromosome region maintenance 1 (CRM1) also known as exportin 1 [124]. Cargo proteins contain sequences known as nuclear localization (NLS) and nuclear export signals (NES). These sequences are recognized by different members of the karyopherin family: respectively importins and exportins. 

Most NLS sequences belong to the classical NLS category, which include monopartite NLS (4–8 amino acids with an abundance of basic Lys and Arg residues [125]) and bipartite NLS which is two clustered sequences of basic amino acids separated by 10–12 unconserved residues (KR-10aa-KKKL is typical) [126,127]. Non-classical NLSs have no obvious features in the amino acid sequence and do not contain clustered basic amino acid residues [128,129]. Whereas classical NLS sequences require importin α for their function, non-classical NLSs can directly bind to importin β in the absence of importin α [130]. Most nuclear export sequences are hydrophobic leucine-rich domains [131,132].

Sequence analysis identified two NLS sequences in Gli1, which are conserved in Drosophila Ci and in Gli2/3: NLS1 (amino acids 79–84), and NLS2 (amino acids 383–401aa; Figure 1) [133,134]. NLS2 is a classical bipartite NLS, but NLS1 has features of both a classical monopartite NLS, which binds to the importin α/β1 complex [133,135] and of non-classical proline-tyrosine (PY) NLS, which cooperates with importin β2 independently of importin α [112,136]. Both NLS sequences are involved in Gli protein nuclear import via importin α/β1 [112,133,135,137], with NLS2 playing a more dominant role [112,133]. The role of importin β2 in nuclear import of Gli proteins via the PY-NLS features of NLS1 is less clear. In an artificial system blocking importin β2 inhibits the nuclear import of Gli2/3 fragments that contain NLS1 [136]. However, importazole-sensitive transport via importin α/β1 appears to be significantly more important for nuclear accumulation of Gli proteins expressed at endogenous levels than M9M-sensitive transport via importin β2/PY-NLS [135]. Moreover, any effects of importin β2 on Gli protein nuclear accumulation may be indirect and caused by the fact that importin β2/PY-NLS is crucial for the accumulation of Gli proteins in the primary cilium [112,135].

In addition to NLS sequences, all three Gli proteins contain leucine-rich nuclear export signals, but their sequences are not strongly conserved among members of the Gli family. Substitution of two evolutionary leucine residues within the Gli1 NES (aa 495–513) causes the N-terminal fragment of Gli1 to be located only in the nucleus, and so does inhibition of exportin 1 with leptomycin B [133,138]. Another leucine-rich motif (887–895 in the mouse Gli2, conserved in Gli3) works similar to the NES motif of the Gli1 protein [133]. The poor conservation of NES signals in contrast to the highly conserved NLSs suggests that Gli protein nuclear export may be dependent on structural motifs rather than simple primary sequence [139] or that novel motifs and/or unconventional exportins are involved. 

As with ciliary transport of Gli proteins, their nucleocytoplasmic shuttling is highly regulated. PKA determines the ability of Gli proteins to enter the nucleus in several ways. First, importin β1 binding to Gli1 is inhibited by the PKA-mediated phosphorylation of a threonine near NLS2 [47]. This threonine is present also in Gli2 (Thr556) but it was never found to be phosphorylated [140]. Second, PKA was shown to induce the binding of Gli proteins to 14-3-3ε, which traps them in the cytoplasm [141], although this has been contested in later studies [23]. Third, it may regulate Gli nuclear localization indirectly by inhibiting its accumulation in the primary cilium and dissociation from SuFu.

SuFu is a strong negative regulator of Gli nuclear accumulation. Binding of SuFu to a region that contains an SYGH motif has been shown to prevent importin β1 and β2 from binding to NLS1 [133,136,137,138]. Accordingly, Gli proteins are primarily nuclear in SuFu-depleted cells [19]. Interestingly, SuFu was also found to chaperone Gli proteins into the nucleus and to bind chromatin in conjunction with them, suggesting that the SuFu-Gli interaction may have a bi-functional role in Gli protein nucleocytoplasmic transport [98].

Until recently, most researchers have assumed that ciliary transport of Gli proteins is essential for their activation and translocation into the cell nucleus. However, there are reasons to suggest that the Gli proteins do not need cilia to reach the nucleus. It was shown that the Gli2 protein lacking the N-terminal repressor domain can induce skin tumors even in the absence of primary cilia [142], suggesting that it must enter the cell nucleus without the involvement of cilia. The issue of the dependence or independence of nuclear transport of Gli proteins from primary cilia is significant because in many cancers primary cilia disappear [143], and therefore therapies targeting processes dependent on ciliary transport may prove ineffective.

## 6. Mechanisms of Transcriptional Activation by Gli Proteins

Of the three mammalian Gli proteins, Gli1 appears to exclusively activate target gene transcription, whereas Gli2 and Gli3 can act as transcriptional activators (full-length, primarily Gli2) or C-terminally truncated repressors (mostly Gli3). The transcriptional activation of Gli proteins is mediated through their C-terminal part located downstream of the DNA-binding Zinc finger domain. Early work identified two activator domains, known as A1 and A2, corresponding to amino acids 642–1183 and 1184–1544, respectively, in mouse Gli2 [44]. Specifically, the C-terminal end (amino acids 1451–1544) of the A2 domain was shown as critical to counteract the repressive activity of the N-terminal Gli2 domain. Several protein complexes have been proposed as candidate co-activators of Gli proteins that are recruited by the full-length Gli activators to target promoters/enhancers and induce target gene transcription.

A large group of transcription factors induces target genes by binding the general transcription factor complex TFIID, which in turn directly recruits RNA Polymerase II (PolII) to the target promoters. The Taf9/TafII-31 subunit of TFIID binds to Gli1 and Gli2, but not Gli3. Abrogation of this binding by a point mutation decreases the potency of Gli1 as a transcriptional activator [144,145]. Interestingly, the putative Taf9-binding domain of Gli1/2 is localized in the vicinity of the C-terminus within the strongly activating subdomain of A2 previously identified in Gli2 (amino acids 1451–1544), suggesting that TFIID recruitment is a major mode of activation of gene transcription by Gli factors. Importantly, mutation of the critical amino acids that mediate the interaction of Gli1 with Taf9 does not completely abrogate its ability to induce target genes but rather weakens it [144], suggesting that additional factors play a role in this process. 

Another known PolII-recruiting complex is the mediator. Gli3 interacts with several subunits of the mediator complex, including Med1, Med12, Med14, Med23, and CDK8 via a domain that corresponds to the C-terminal part of A1 and the whole A2 domain (corresponding to amino acids 1090–1583 of mouse Gli3, or 1045–1544 for mouse Gli2) [146]. It appears that different subcomplexes of the Mediator either induce or repress the transcriptional activity of Gli proteins, with the Med12 subcomplex playing an inhibitory role [146,147].

PolII transcription initiation and elongation can additionally be regulated by the Paf1 complex. Parafibromin/Hprt2, a component of the Paf1 complex, binds the N-terminal conserved region of Gli proteins corresponding to amino acids 279–348 in mouse and human Gli3 and is involved in Gli1/2-mediated transcriptional activation [148]. Interestingly, the Hprt2-binding region of Gli proteins overlaps with the PY nuclear localization sequence and the SuFu binding site.

Histone acetylation at target promoters is an important hallmark of gene activation. The Cbp/Ep300 family of coactivators regulates transcription both by direct recruitment of PolII to target promoters and by acetylating histones and promoting open chromatin conformation. CBP binds both domain A1 [149] (amino acids 827–1132) and domain A2 [146] (amino acids 1090–1596) of Gli3, and was shown to enhance transcriptional activation by Gli3, but not Gli1. Its role in Gli2 activity has not been studied so far. Interestingly, symptoms caused by a mutation in *CBP* resemble those of Greig cephalopolysyndactyly [150], a disease associated with mutations of the *GLI3* gene [151]. 

In a more recent screen of mammalian histone acetyltransferases, Ep300, but not Cbp, was found to be required for receptor-mediated induction of Gli-protein-mediated transcription [90], suggesting that the role of Cbp in Gli activity may be more nuanced. The same study identified the histone acetyltransferase Pcaf as a major coactivator of Gli proteins. Pcaf physically interacts with Gli1, and its depletion reduces the expression of Hh target genes and the acetylation of target gene promoters [90]. Interestingly, Pcaf was also found to play a role in Gli1 ubiquitination and destabilization in a manner independent of its acetyltransferase activity, suggesting that it may play a dual role in Gli protein regulation [89]. The acetylated histone-binding BET bromodomain protein Brd4 is required for the induction of at least some Hh target genes [152,153], which may explain why histone acetylation is an important epigenetic modification that mediates Gli activity.

In addition to histone acetylation, a major chromatin modification pathway is histone methylation at histone 3 lysine 9 (H3K9) and lysine 27 (H3K27). H3K27 trimethylation (H3K27me3) is a major repressive epigenetic signal. Upon activation, Gli2 and Gli1 bind to the histone demethylase Jmjd3, which reduces H3K27me3 levels at Gli target gene promoters and increases the expression of target genes [154].

The induction of many genes requires the ATP-dependent repositioning of nucleosomes. The Swi/Snf complex is a major player in ATP-dependent chromatin remodeling and plays an important role in Gli-mediated transcription. The ATPase Brg1/Smarca4, which is the catalytic component of Swi/Snf, has a dual function—it binds to the repressor and activator domains of Gli1/2/3 and cooperates with Gli3R in repressing Hh target genes, as well as with Gli1/2A in activating them [155]. Brg1’s functions both as a repressor and activator appear to be independent of its ATPase activity, but may depend on the ability of the complex to recruit histone deacetylase HDAC2 to target promoters. As co-repressor, the Brg1/HDAC2 complex may regulate target genes through deacetylation of histones at target gene promoters, but the co-activator function is likely to involve HDAC targets other than histones, such as the Gli1/2 transcription factors themselves [24,155]. The coactivator function of Brg1 appears to be dominant over the corepressor function, at least in cerebellar development and medulloblastoma [156]. 

A different component of the Swi/Snf complex, Srg3/Smarcc1, plays a key role in Gli2/3-dependent transcription during limb development [157]. Deletion of Srg3 both derepresses target genes typically inhibited by Gli3R and prevents full activation of GliA-regulated genes, which is in line with the bifunctional role of the Swi/Snf complex.

In addition to traditional co-activators and chromatin remodeling factors, a novel protein Mim/Beg4 was shown to enhance Gli1-mediated transcription and bind to the Gli1/SuFu complex [158]. It is however, unclear, whether the effect on Gli-mediated transcription is direct, or is mediated through the involvement of Mim in the regulation of ciliogenesis [159].

## 7. Mechanisms of Transcriptional Repression by Gli Proteins

In the absence of upstream signal, Gli3, and to a lesser degree Gli2, are proteolytically truncated and play the role of transcriptional repressors (GliR) [160]. GliR-dependent repression is mediated by the N-terminal domain of Gli2 and Gli3 [44]. The N-terminal domain can be subdivided into the bona-fide repressor domain and the downstream SuFu-binding domain. In the absence of Hh ligand, SuFu antagonizes Gli-dependent transcription in several ways; it limits the translocation of full-length Gli proteins into the nucleus [19] and facilitates Gli3R formation by recruiting GSK3β [161], but also binds to Gli proteins inside the nucleus and recruits transcriptional co-repressors to shut down target gene expression. Importantly, Gli3R is capable of inhibiting transcription also independently of SuFu via its intrinsically disordered repressor domain [162,163], but the mechanism of SuFu-independent transcriptional repression by Gli3R has not been fully elucidated.

The best-studied function of the N-terminal domain is to recruit histone deacetylases (HDAC) [24,164]. One of the major HDAC-dependent co-repressor complexes is the Sin3 complex, which mediates histone deacetylation through HDAC1 and HDAC2. SAP18, a component of the Sin3 HDAC complex, was shown to interact with the N-terminal domain of Gli proteins via SuFu. Gli1, SuFu, SAP18 and Sin3 are capable of forming a complex on DNA [165]. The interaction between GliR/SuFu and the Sin3 complex may be indirect. Ski and the related Sno protein act as corepressors and directly bind to the Sin3 complex and another co-repressor complex known as N-CoR/SMRT. Ski binds to the N-terminal domain of Gli3 and Gli2, but not Gli1. Antagonizing Ski function via RNAi or anti-Ski antibodies impairs Gli3R-dependent transcriptional repression suggesting that Sin3 binding to Gli3R/SuFu may depend on Ski [164]. 

In addition to the Sin3 complex, HDAC can be recruited to DNA via the NuRD (nucleosome remodeling and histone deacetylase) repressor complex. p66β, a component of the NuRD complex, as well as HDAC1, are recruited to Gli2 and Gli3 via SuFu to inhibit transcription of target genes. Interestingly, p66β binds full-length, but not truncated repressor forms of Gli2 and Gli3, suggesting that it is not a major mediator of Gli3R-dependent transcriptional repression [97].

The Swi/Snf chromatin remodeling complex plays a dual coactivator/corepressor role in Gli-mediated transcription. Unlike Brg1, which is primarily a coactivator (see Section 6), Snf5, a different component of the Swi/Snf complex, acts exclusively as a Gli co-repressor. Snf5, as well as Baf57/Smarce1 and Baf170/Smarcc2 were identified as binding partners of Gli1. Loss of Snf5 leads to the activation of the Hh pathway, whereas its expression in the Gli1-dependent malignant rhabdoid tumors suppresses Gli1 activity and tumor growth [97,166].

Interestingly, of the co-repressors mentioned, none appear specific for Gli3R. Given the fact that loss of Gli3R is associated with severe developmental defects that are not compensated for by Gli2 or Gli1, we may speculate that some Gli3R-associated co-repressor proteins remain undiscovered. Future work should shed more light on GliR-dependent transcriptional regulation and on the interplay between the different modes of Gli-mediated activation and repression of Hh target genes in development and disease.

## 8. Gli Proteins in Cancer

Aberrant activation of the Hh-Gli pathway in adult cells may contribute to carcinogenesis or accelerate the course of tumor growth by enhancing cancer cell proliferation, survival, stemness, metastatic potential, or by stimulation of stroma and blood vessel formation. There are fundamentally three different mechanisms that result in aberrant constitutive activation of Hh-Gli pathway. The first mechanism entails mutations of Hh component genes (missense/nonsense/frameshift mutations or copy number variations). The second mechanism consists of overexpression or silencing of pathway components and Gli-interacting proteins, often through non-canonical mechanisms or via aberrant microRNA. The third mechanism depends on the posttranscriptional modification of Gli1/2 mRNA through splicing and RNA editing. 

In the absence of genetic or epigenetic alterations at the level of the canonical Hh pathway components, overactivation of Gli proteins may occur non-canonically via cross-talk with other signaling pathways. Among pro-tumorigenic pathways that regulate Gli proteins independently of the canonical Ptch1-Smo axis are: the mitogen-activated kinases (MAPK) cascade, the transforming growth factor beta (TGFβ) pathway, the epidermal growth factor receptor (EGFR) pathway, and the fibroblast growth factor receptor (FGFR) pathway [167,168,169]. Conversely, in some cases, Hh signaling via the Hh-Ptch1-Smo axis may also induce tumor formation independently of Gli proteins [170].

Gli proteins induce and support tumorigenesis by regulating the transcription of various pro-oncogenic factors. For example, cell proliferation in Gli-related cancers is enhanced by Gli-dependent expression of cyclins D1 and D2 [171] or the N-myc proto-oncogene [172]. Gli proteins regulate Sox2 and Nanog expression to mediate self-renewal of cancer stem cells [173,174]. Epithelial to mesenchymal transition (EMT) might be initiated by transcription of Snail [175]. Gli-dependent angiogenesis can be stimulated by upregulation of angiopoietins and vascular endothelial growth factor (VEGF) [176,177]. Gli proteins can protect cancer cells from apoptosis by inducing Bcl2 [178]. Finally, Gli proteins may help tumors evade the immune system by stimulating the expression of TGFβ2 [179]. In a number of cancers, Gli proteins are activated not in the transformed cells themselves, but rather in the tumor stroma, promoting paracrine positive feedback loops [180,181]. 

Three recent reviews have addressed currently available and potential future therapeutic interventions that target Gli proteins in cancer [40,42,182]. In the current review we list the most salient discoveries of the roles of Gli proteins in oncogenesis based on in vitro and in vivo loss-of-function studies [183,184,185,186,187,188,189,190,191,192,193,194,195,196,197,198,199,200,201,202,203,204,205,206,207,208,209,210,211,212,213,214,215,216,217,218,219] (Table 2). In some cancer types there is evidence of genetic and epigenetic alterations of genes encoding Gli proteins, but the data is correlative rather than functional [220,221,222,223,224,225,226,227,228,229,230,231,232,233,234]. We list some of the recent studies in that category in Supplemental Table S1. A more comprehensive list of mutations of *GLI1/2/3* genes can be found in online databases such as COSMIC (https://cancer.sanger.ac.uk/cosmic) and AACR Project GENIE (http://synapse.org/genie).

## 9. Gli Proteins in Development and Tissue Regeneration

The development of multicellular eukaryotes is driven by the interplay of cell proliferation and differentiation. The Hh/Gli pathway plays a key role both in stimulating cell divisions in undifferentiated progenitor cells and in committing cells to a specific fate. Not all three Gli proteins are involved in the Hh-dependent development of each tissue. Specifically, Gli1 appears to be, to a large degree, dispensable for normal mammalian development [235,236,237], while the relative importance of Gli2 and Gli3 varies from tissue to tissue. *Gli2* null mice die before birth with defects in various tissues including the central nervous system, skeleton, and lungs [237,238,239,240,241]. Interestingly, when *Gli1* is expressed from the *Gli2* locus, it can substitute for the latter in most developmental processes, suggesting that Gli2 is a bona fide activator throughout development [242,243]. *Gli3* null mice die perinatally and are characterized by severe craniofacial defects, central nervous system malformations, and polydactyly [244]. Unlike *Gli2*, *Gli3* is haploinsufficient: *Gli3* heterozygotes are viable, but have various developmental malformations, such as craniofacial defects and polydactyly [238,240,241,244,245,246]. Mice expressing constitutive Gli3R instead of full-length Gli3 from both alleles are viable at birth, but die before weaning, due to various developmental defects at the level of the skeleton, digestive tract, upper respiratory tract, kidneys, and adrenal glands [247].

Both Gli2 and Gli3 are involved in the patterning of the neural tube. In the ventral neural tube, high concentrations of Shh secreted from the notochord promote the creation of GliA and block the formation of Gli3R. Gli2/3A steers neural progenitors towards ventral cell fates (floor plate, ventral interneurons) and suppresses the expression of dorsal markers in the spinal cord [23,248]. Interestingly, Gli2A is crucial for the development of ventral cell types (floor plate, V3 interneurons) in the caudal (trunk and tail) region of the spinal cord, while Gli3A is sufficient to induce ventral cell fates in the head and thorax [238,241]. Intermediate cell fates (V0, V1, V2 interneurons) are controlled through the antagonism between Shh ligand and Gli3R. Specifically, experiments performed on embryos deficient in *Gli3* and either *Shh* or *Smo* indicate that Gli3 is required and sufficient for the repression of V0–V2 interneuron markers in the absence of upstream signal [249,250]. Both GliA and GliR are important for motor neuron specification [249,251,252]. Unlike in the spinal cord, patterning of the telencephalon is partially independent of Hh/Gli, and Gli3R plays a permissive rather than instructive role in this region [253]

Apart from the neural tube, the role of Hh/Gli in organ patterning is best studied in the limbs [254]. The Shh ligand is expressed in posterior limb buds (so called zone of polarizing activity) and induces the formation of gradient of GliA and GliR throughout the antero-posterior axis [255]. The number and identities of digits are determined by the interplay of GliA and GliR, with Gli3R playing a major patterning role in the anterior limb, and Gli2/3A contributing to the development of posterior digit identities [243,244,245,247,256,257]. This is very reminiscent of neural tube patterning, where effects mediated by Gli3R prevail in tissues positioned farther away from the Shh source, and Gli2/3A plays a more important role closer to the source of the morphogen (the notochord in the neural tube and the zone of polarizing activity in the limb). 

The interpretation of GliA/GliR gradient in tissue patterning is mediated through various Gli binding motifs positioned in promoters and enhancers of developmentally-relevant genes and occurs in a timing-dependent manner in collaboration with other transcription factors, such as SoxB1 [251,258].

The Hh pathway is critical for normal bone development and repair [259,260]. Rather than Shh, bone formation is controlled by Indian hedgehog (Ihh). Endochondral (long bone) ossification is well known to be dependent on Ihh, but the role of Hh/Gli in intramembranous ossification is less well established. Ihh regulates bone formation and repair via all three Gli proteins. *Gli2* mutant mice display various defects in bone development in the craniofacial skeleton, vertebrae, long bones of the limb (stylopod and zeugopod), and sternum [240]. Mechanistically, Gli2 is required for the induction of cartilage vascularization—an essential step in the conversion from immature cartilaginous bone to mature mineralized tissue [261,262]. On the other hand, inhibition of Gli3R formation appears crucial for the induction of chondrocyte proliferation and hypertrophy [261,263,264], while both derepression of Gli3R signal and full activation of Gli2A are involved in osteoblast differentiation by inducing the osteoblast-specific transcription factor Runx2 [261,265]. Consequently, induction of Gli2A and inhibition of Gli3R formation by inhibiting PKA activity induces ectopic ossification in the joints [266]. Although adult *Gli1* mutant mice appear to have normal bones, analyses of *Gli1* KO fetuses showed that the early development of osteoblasts requires Gli1 as well as Gli2 and Gli3 [267]. Importantly, Gli1 is also a marker for early osteoblast progenitors both during development and during repair of fractured bone [259]. 

In contrast to endochondral ossification, which is clearly affected by impairment of Hh/Gli signaling, intramembranous ossification phenotypes of Gli mutant mice, mostly affecting bones of the skull, are more subtle. Loss of *Gli3* induces abnormal development of the calvaria characterized by premature ossification of the interfrontal suture (craniosynostosis) caused by abnormal osteoprogenitor proliferation and differentiation [268,269].

In the central nervous system, the Hh pathway is not only a major regulator of neural tube patterning, but also plays additional roles during the creation of mid-hindbrain and in the growth and patterning of the cerebellum. Gli2A and Gli1 control ventral neuron specification in the mid-hindbrain before mid-gestation, as well as cerebellar foliation and expansion of the granule cell population in the cerebellum at later stages [270,271,272]. On the other hand, Gli3R controls the overall levels of growth of the mid-hindbrain region and suppresses the expression of the anteroposterior organizer Fgf8 [270]. Moreover, Gli3R can rescue most of the phenotypes caused by overactive Gli signaling due to targeted loss of *SuFu* in the mid-hindbrain. These phenotypes include delayed differentiation of cerebellar cells and severe malformations at the level of the cerebellum and hindbrain structures [273].

There are many other organs and tissues that rely on Gli proteins for their development. In the excretory system, Gli3R controls branching morphogenesis in the kidney, nephron number, and ureter pacemaker cells [274,275,276], and Gli2 represses smooth muscle differentiation in bladder mesenchyme via BMP [277]. In the sclerotome Gli2 and Gli3 plays both activating and repressive roles [278]. In the respiratory system, Gli3 affects the size and shape of the lung lobes [279,280] and Gli2/3 interact to control respiratory tract development [280]. In the placenta, Gli2 and Gli3 are important for the development of the placental labyrinth [281]. In the mammary, Gli3R is required for induction and patterning of mammary epithelium and bud formation [282,283] and Gli2 is required in the stroma for proper duct formation [284]. Constitutive Gli3R expression causes malformation of the digestive system, including imperforate anus [247]. Gli1 and Gli2 play redundant activating roles in the maturation of Leydig cells in the testis [285]. Loss of *Gli2* causes growth arrest in the hair follicle [286]. Gli1, Gli2, and Gli3 play non-redundant roles in T-cell maturation in the thymus and B-cell development in the liver [236,287,288,289]. Gli2, and to a lesser degree Gli1, are involved in pituitary gland formation, and deletion of both genes results in complete loss of the pituitary gland [237]. During skeletal muscle development both Gli2A and Gli3R play a role in somite patterning [290]. Early in development, Gli2 is also involved in the anteroposterior patterning of the mesoderm [291]. Other tissues, such as the prostate, express Gli proteins throughout development, but the role of Gli1/2/3 in their development has not been explicitly studied or was found to be inconclusive [292].

Developmental processes controlled by Gli proteins become evident in diseases associated with mutations of genes encoding these proteins. Non-synonymous heterozygous *GLI2* mutations underlie a significant fraction of congenital hypopituitarism and also cause holoprosencephaly-like malformations, including abnormal craniofacial development, growth retardation, and microcephaly [293,294,295]. *GLI3* mutations were found to be involved in two developmental syndromes: Pallister-Hall syndrome and Greig cephalopolysyndactyly syndrome [296,297]. Greig syndrome, and its more severe form known as acrocallosal syndrome, is associated primarily with loss-of-function heterozygous mutations of *GLI3*. Its symptoms resemble those of *Gli3* heterozygous mice (*Gli3*^XtJ^, the extra-toes mouse) and suggest that, as in mice, *Gli3* is haploinsufficient in humans [244]. On the other hand, Pallister-Hall syndrome is a result of heterozygous dominant active mutations that produce a truncated Gli3 that functions as a constitutive repressor. Accordingly, Pallister-Hall symptoms are mirrored to a large degree by a mouse model with constitutive Gli3R expression (*Gli3*^Δ699^) [253]. However, unlike in humans, most Pallister-Hall symptoms in mice only become apparent when both alleles of *Gli3* are replaced with *Gli3^Δ699^* [247]. In contrast to *GLI2* and *GLI3*, where human mutation symptoms are similar to mouse knockout phenotypes, mutations of *GLI1* cause more severe phenotypes in humans than in mice [237]. Homozygous truncating *GLI1* mutations were recently found to cause developmental defects whose symptoms, such as postaxial polydactyly, mental retardation, and shortened lower limbs, overlap with those of Ellis-van Creveld syndrome [298]. These phenotypes are absent in mouse *Gli1* knockouts [237]. A more comprehensive review of human birth defects associated with Gli protein mutations is provided in [42]. 

Besides embryonic development, Gli proteins are also key in postnatal regeneration and adult stem cell maintenance. Gli proteins are expressed in postnatal neural progenitors in the brain, and interference with Shh signaling results in the impaired generation of new neurons in the olfactory bulb of adult mice mostly by promoting constitutive Gli3R formation [299,300]. In the postnatal retina, neural progenitors and Müller glia are regulated by the interplay of Hh and Notch pathways in a manner dependent on Gli2 [301]. In the breast, Gli2 expressed in the stroma supports the self-renewal of mammary stem cells [302]. Gli1 is involved in postnatal hematopoiesis, regulating hematopoietic stem cells and myeloid progenitors [303] as well as T-cell maturation [236]. Gli1 also appears to play a role in postnatal regeneration of the heart and proliferation of adult cardiomyocytes [304]. Regeneration and stem cell pool maintenance in several other tissues was shown to be dependent on Hh signaling, but the role of specific Gli transcription factors was not studied [305].

## 10. Summary

Gli proteins are central to many developmental and disease processes in humans and other vertebrates. Their complex regulation is accomplished both by canonical Hh signaling and by non-canonical cross-talk with other pathways. Many processes, such as posttranslational modifications, intracellular transport, and interactions with a plethora of binding partners, converge on fine-tuning of the balance between Gli activators and Gli repressors. This balance, in turn, governs the expression of Hh target genes, and in consequence guides development, tissue regeneration, and disease processes. 

Out of the three Gli proteins expressed in mammals, Gli3 appears to play almost exclusively the role of a repressor, even though it is equipped with a C-terminal activator domain homologous to that of Gli2. The two most likely reasons are: (1) the strong repressive activity of the N-terminus of Gli3, which likely counteracts its GliA function; and (2) its capacity to be C-terminally truncated by the proteasome. Conversely, Gli2 seems to be primarily an activator, despite possessing a repressor domain, because it is poorly converted to a C-terminally truncated GliR form. The interplay between Gli3R and Gli2A determines the outcome of Hh signaling during development, with Gli1 playing only a secondary role. The regulation of Gli2 and Gli3 is mostly mediated by their posttranslational modifications. Specifically, phosphorylation by protein kinase A functions as a master switch between GliA and GliR. In contrast, the activity of Gli1, which is an exclusive activator, is to a large degree dependent on its expression level. Gli1 expression is fine-tuned by the balance between Gli2A and Gli3R, but can also be regulated by cross-talk with other signaling pathways, especially TGFβ and Ras/MAPK. Interestingly, Gli1 seems to play a more prominent role in cancer than Gli2 or Gli3, at least based on the number of published studies. It is tempting to speculate that this may be because Gli1 is less sensitive to inhibitory posttranslational modifications, such as PKA phosphorylation, or because its expression is more flexible, enabling cancer cells to more easily hijack it to promote their survival and proliferation.

Although we now know much about the biochemistry of Gli proteins and their roles in physiology and pathology, several questions remain unresolved:

1. How do the different posttranslational modifications of Gli proteins interact with one another? Which modifications coexist, and which are mutually exclusive?
2. What are the kinases and phosphorylation sites responsible for the phosphorylation of active nuclear Gli proteins?
3. What is the importance of Cul3/Spop for degradation of Gli3R and Gli2?
4. What are the proteins that regulate the accumulation of Gli proteins at the base of the primary cilium prior to crossing the ciliary barrier?
5. What are the proteins that participate in nuclear and/or ciliary export of Gli proteins?
6. What happens to Gli proteins at the tip of the primary cilium that makes them active?
7. In what circumstances do the different transcriptional co-activators and co-repressors interact with nuclear Gli proteins? Which of these co-activators and co-repressors play roles in regulation of specific target genes?
8. What is the functional significance of mutations in Gli proteins that occur in cancer?

Answering these questions will not only help us understand the biology of Gli proteins better, but may also reveal novel therapeutic approaches that we can use in Gli-dependent disease states.

## Figures and Tables

**Table 1:** Posttranslational modifications of Gli proteins.

Type *	Enzyme	Residue Number						Effect on the Hh Pathway	Reference
		mGli1	hGli1	mGli2	hGli2	mGli3	hGli3		
P	PKA			S789, S805, S817, S848, S923, S956		S849, S865, S877, S907, S980, S1006		inhibition, increased processing to GliR, reduced nuclear accumulation of Gli2/3	[23]
P	PKA		T374					inhibition due to decreased nuclear localization	[47]
P	PKA		S544, S560					inhibition	[48]
P	aPKC	S246 **, S307 **	S243, S304					activation, increased DNA binding, promoting deacetylation	[49,50]
P	AMPK	S105 **, T1079 **	S102, S408, T1074					inhibition, destabilization	[51]
P	MEKK1		+ ***					inhibition	[52]
P	Hck		Y800					activation	[53]
P	GSK3β						S861, S873, S903	inhibition, increased GliR formation	[46]
P	?						S850, S894	inhibition, increased GliR formation	[46]
P	GSK3β			S801, S813, S844				inhibition, destabilization	[54]
P	CK1						S852, S868, S880, S910	inhibition, increased GliR formation	[55]
P	CK1			S792, S808, S820 S851				inhibition, destabilization	[54]
P	Dyrk1a		+ ***					activation, increased nuclear accumulation	[56]
P	Dyrk1b		+ ***					non-canonical activation due to AKT phosphorylation	[57]
P	Dyrk2			S385, S1011				inhibition, destabilization	[58]
P	ULK3	+ ***		+ ***		+ ***		activation	[59]
P	S6K1	S87 **	S84	-		-		non-canonical activation	[60]
P	AKT	-		S230	S234 **	-		non-canonical activation, stabilization	[61,62]
P	CIT			S145 **	S149			non-canonical activation, nuclear accumulation	[63]
P	?			S248				activation (?)	[23]
P	?	-		S662		-		inhibition, destabilization	[64]
Ub	Cul1/β-TrCP					K773, K779, K784, K800		inhibition, GliR formation	[46]
Ub	Cul3/Spop				+ ***		+ ***	inhibition, destabilization	[65]
de-Ub	HAUSP		+ ***		+ ***		+ ***	activation, stabilization	[66]
de-Ub	USP48		+ ***					activation, stabilization	[67]
SUMO	Pias1		K180, K815	K376, K630, K716			K87, K462, K696, K779	activation, stabilization	[68]
SUMO	?			K630, K716				inhibition, binding to HDAC5 (?)	[69]
de-SUMO	SENP1	K180, K415, K815						inhibition, destabilization, nuclear export	[70]
Ac	p300, HATs		K518					inhibition	[24]
de-Ac	HDAC1		K518					activation	[24]
G	?		+ ***		+ ***			activation, nuclear accumulation	[71]
Me	Set7					K436, K595		stabilization, increased DNA binding	[72]

* Modification types: P—phosphorylation, Ac—acetylation, Ub—ubiquitination, SUMO—sumoylation, G—O-GlcNAcylation, Me—methylation; ** by homology; *** residue unknown; ? – enzyme unknown; hGli—human Gli, mGli—mouse Gli.

**Table 2:** Functional significance of Gli proteins in cancer based on loss-of-function studies.

Member of Gli Family	Tumor Type	Experimental Model	Activation Cause	Effect	Reference
**Canonical Activation via Hh/Ptch1/Smo**					
Gli1	melanoma	primary cell culture, short term culture xenograft	Smo-dependent	cancer stem cell self-renewal, tumor initiation	[183]
Gli1, Gli2	medulloblastoma	genetic mouse model	Ptch1 mutation	malignant transformation	[184]
Gli2	medulloblastoma	cell culture	Smo-dependent	proliferation, viability	[185]
Gli1	colon cancer	primary cell culture, short term culture xenograft	Smo-dependent	cancer stem cell self-renewal, proliferation, xenograft growth, metastasis	[186]
Gli1, Gli2	glioma	primary cell culture	Smo-dependent	cancer stem cell self-renewal, proliferation, viability	[187]
Gli1	pancreatic and colon cancer	cell co-culture, patient-derived xenograft	Hh ligand secretion	stroma induction	[188]
Gli1	prostate cancer	cell co-culture, cell line xenograft	Hh ligand secretion	stroma induction	[180]
?	skin cancer (BCC-like)	genetic mouse model	mutant Smo	immune inhibition via TGFβ	[179]
Gli1, Gli2	basal cell carcinoma	cell culture	Smo-dependent	synergy with EGFR/AP-1, malignant transformation, viability	[189]
Gli1, Gli2	breast cancer	cell culture	Smo-dependent	p53 inhibition through activation of Mdm2	[190]
**Overexpression of Gli Proteins/Aberrant microRNA Silencing**					
Gli1, Gli2	pancreatic cancer	cell culture	Gli protein overexpression	migration, invasion, DNA damage	[191,192]
Gli1	non-small cell lung cancer	cell culture	Gli1/Gli2 overexpression	cancer stem cell self-renewal	[173]
Gli1	rhabdomyosarcoma	cell culture, CAM assay	loss of *GLI1* antisense RNA	proliferation, tumor growth in CAM assay	[193]
Gli1	medulloblastoma	cell culture	loss of *miR-324-5p*, also regulates Smo	proliferation, colony growth on soft agar	[194]
Gli1	glioma	cell culture	loss of *miR-218*	migration, invasion	[195]
Gli1	gastric cancer	cell culture, cell line xenograft	loss of *miR-202-3b* and *miR-133b*	migration, invasion, xenograft growth and metastasis	[196,197]
Gli2	gastric cancer	cell culture	loss of *miR-218*	proliferation, migration, invasion	[198]
Gli2	gastric cancer	cell culture	loss of *miR-202*	proliferation, viability	[199]
**Cross-Talk with Other Pathways**					
Gli2	pancreatic cancer	cell culture	Wnt	c-Myc expression, colony growth in collagen, drug resistance	[200]
Gli1	pancreatic cancer	cell culture, genetic mouse model	TGFβ, KRAS	viability, malignant transformation	[201]
?	Prostate cancer	cell line xenograft	Smo-independent	xenograft growth	[202]
?	breast cancer	cell culture, cell line xenograft	Smo-independent	proliferation, viability, xenograft growth	[203]
Gli1	pancreatic cancer	cell culture	KRAS	growth on soft agar	[204]
Gli1	melanoma	cell line xenograft	NRAS, but also Smo-dependent	xenograft growth, recurrence, metastasis	[205]
Gli2	pancreatic cancer	cell culture	TGFβ	proliferation	[206]
Gli1	esophageal cancer	cell culture, cell line xenograft	TNFα/mTOR, but also Smo-dependent	viability	[60]
Gli1	pancreatic cancer	cell culture	TNFα/IL1β	migration, invasion, EMT and drug resistance	[207]
Gli1	breast cancer	cell culture, cell line xenograft	NFκB	viability, motility, EMT, clonogenicity, self-renewal	[208]
Gli1	melanoma, breast cancer	cell culture, cell line xenograft	WIP1, but also Smo-dependent	proliferation, self-renewal, xenograft growth	[80]
Gli1	glioblastoma	cell culture, cell line xenograft	p53, but also Smo-dependent	feedback loop on p53, proliferation, self-renewal, xenograft growth	[209]
Gli1	Ewing sarcoma family of tumors	cell culture	EWS-FLI1	proliferation, growth on soft agar	[210]
Gli1	Burkitt lymphoma	cell culture	MYC	viability	[211]
Gli2	medulloblastoma	genetic mouse model	ATOH1, but also Smo-dependent	proliferation	[212]
Gli3	colon cancer	cell culture	GSK3B	proliferation, viability	[213]
Gli1	breast cancer	cell culture	loss of *SETD7*, but also Smo-dependent	proliferation, migration, invasion	[214]
Gli3	non-small cell lung cancer	cell culture, cell line xenograft	SETD7	proliferation, xenograft growth and metastasis	[72]
Gli2	breast cancer	cell culture, cell line xenograft	BCAR4 lncRNA	growth on Matrigel, xenograft metastasis	[63]
Gli2	breast cancer	cell culture, cell line xenograft	FOXC1	self-renewal, xenograft growth	[215]
**RNA Splicing or Editing**					
Gli1	glioblastoma	cell culture	tGLI1 splice variant	cell migration, invasion	[216]
Gli1	glioblastoma	cell culture	tGLI1 splice variant	angiogenesis	[176]
Gli1	breast cancer	cell culture	tGLI1 splice variant	angiogenesis, migration, growth on soft agar	[217]
Gli1	medulloblastoma	cell culture	RNA editing	proliferation	[218]
Gli1	multiple myeloma	serial transplant xenograft	RNA editing	proliferation, transplant growth	[219]

?—specific Gli protein unknown.

## Supplementary Materials

The following are available online at http://www.mdpi.com/2073-4409/8/2/147/s1, Table S1: Genetic and epigenetic modifications of Gli genes in human cancer.
