# Supplementary material for: Gli Proteins: Regulation in Development and Cancer
Source: Cells. 2019 Feb 11;8(2):147. doi: 10.3390/cells8020147 (PMC6406693; doi:10.3390/cells8020147)
Supplement: Supplementary file 1 [file cells-08-00147-s001.pdf]

Supplemental Table S1. Genetic and epigenetic modifications of Gli genes in human cancer

Member of Gli family	Tumor type	Mutation type	Predicted effect of the mutation	Reference
GLI1, *GLI2*	medulloblastoma	gene amplification	increased expression	[220]
GLI1	breast cancer, rhabdomyosarcoma	gene amplification	increased expression*	[221–224]
GLI2	oral squamous cell carcinoma	gene amplification	increased expression	[225]
GLI1	pericytoma	gene fusion with *ACTB* gene	increased expression, gain of function (loss of SuFu binding domain)*	[226,227]
GLI1	Merkel cell carcinoma	synonymous substitution	no effect on protein function*	[228]
GLI1, *GLI3*	T-cell acute lymphoblastic leukemia	somatic missense substitutions	altered function ? (located in ZF, SuFu binding, and A1 domains)*	[229]
GLI1	gastric and colorectal cancer	frameshift mutations	loss of function*	[230]
GLI1, *GLI3*	breast and colorectal cancer	somatic mutations	? – mostly located in poorly conserved regions*	[231]
GLI1, *GLI3*	pancreatic cancer	somatic mutations	altered function ? (located in repressor and SuFu binding domains)*	[232]
GLI3	gastric cancer	hypermethylation	reduced expression*	[233]
GLI1	soft tissue sarcoma	truncating and nonsynonymous mutations, gene amplification	specific mutations not described*	[234]

* Effects of mutations not tested experimentally
